# Update on Non‐Biological and RNA‐Based Therapeutics in Chronic Inflammatory Diseases: Precision Medicine Through Small Molecules: An EAACI Position Paper

**DOI:** 10.1111/all.70235

**Published:** 2026-02-07

**Authors:** Franziska Roth‐Walter, Ian M. Adcock, Cristina Benito‐Villalvilla, Rodolfo Bianchini, Leif Bjermer, Gaetano Caramori, Luigi Cari, Kian Fan Chung, Zuzana Diamant, Ibon Eguiluz‐Gracia, Edward F. Knol, Milos Jesenak, Francesca Levi‐Schaffer, Giuseppe Nocentini, Liam O'Mahony, Oscar Palomares, Frank Redegeld, Milena Sokolowska, Betty Van Esch, Marco Zurlo, Cristiana Stellato

**Affiliations:** ^1^ Department of Biological Sciences and Pathobiology University of Veterinary Medicine Vienna Vienna Austria; ^2^ National Heart & Lung Institute Imperial College London London UK; ^3^ Department of Biochemistry and Molecular Biology, School of Chemistry Complutense University of Madrid Madrid Spain; ^4^ Department of Biochemistry and Molecular Biology, School of Medicine Complutense University of Madrid Madrid Spain; ^5^ Department of Respiratory Medicine and Allergology, Lung and Allergy Research, Allergy, Asthma and COPD Competence Center Lund University Lund Sweden; ^6^ Department of Medicine and Surgery, Pulmonology University of Parma Parma Italy; ^7^ Department of Medicine, Section of Pharmacology University of Perugia Perugia Italy; ^8^ Experimental Studies Medicine National Heart & Lung Institute, Imperial College London & Royal Brompton & Harefield Hospital, Guys' and St Thomas' NHS Foundation Trust London UK; ^9^ Department of Clinical Pharmacy and Pharmacology University Groningen, University Medical Center Groningen Groningen the Netherlands; ^10^ Department of Respiratory Medicine, First Faculty of Medicine Charles University and Thomayer Hospital Prague Czech Republic; ^11^ Allergy and Clinical Immunology Research Group, Department of Microbiology, Immunology & Transplantation KU Leuven Leuven Belgium; ^12^ Allergy Unit Hospital Regional Universitario de Malaga and IBIMA‐Plataforma BIONAND, RICORS Inflammatory Diseases Malaga Spain; ^13^ Department of Immunology and Dermatology/Allergology University Medical Center Utrecht Utrecht the Netherlands; ^14^ Department of Paediatrics, Department of Clinical Immunology and Medical Genetics Comenius University in Bratislava, Jessenius Faculty of Medicine in Martin, University Teaching Hospital Martin Slovakia; ^15^ Pharmacology & Experimental Therapeutics Unit, Institute for Drug Research, School of Pharmacy, Faculty of Medicine The Hebrew University of Jerusalem Jerusalem Israel; ^16^ APC Microbiome Ireland University College Cork Cork Ireland; ^17^ Department of Medicine University College Cork Cork Ireland; ^18^ School of Microbiology University College Cork Cork Ireland; ^19^ Division of Pharmacology, Utrecht Institute for Pharmaceutical Sciences, Faculty of Science Utrecht University Utrecht the Netherlands; ^20^ Swiss Institute of Allergy and Asthma Research (SIAF) University of Zürich Davos Switzerland; ^21^ Department of Medicine, Surgery and Dentistry “Scuola Medica Salernitana” University of Salerno Salerno Italy

**Keywords:** biomarker‐driven drug development, chronic inflammatory diseases, kinase inhibitors, precision medicine, RNA‐based therapies, small molecule drugs

## Abstract

In the last decades, critical advancements in research technology and knowledge on disease mechanisms steered therapeutic approaches for chronic inflammatory diseases towards unprecedented target specificity. For allergic and chronic lung diseases, biologic drugs pioneered this goal, acquiring on the way—through the clinical use of monoclonal antibodies—a deeper understanding of how inflammatory and immune pathways are configured in disease‐specific patterns. In this biomarker‐driven approach, synthetic small molecule drugs (SMDs) were perceived as lagging behind in innovation for their relative lack of specificity. This was, however, mostly due to a shift in focus towards biologics rather than true obsolescence of SMDs. In the same timeframe, in fact, advances in structural biology and medicinal chemistry, bioinformatics and artificial intelligence held steadily SMDs' innovation and relevance. The use of kinase inhibitors, well established in the treatment of cancer and rheumatological diseases, is now approved for some allergic skin diseases and is approaching asthma and COPD with several clinical trials; moreover, new therapeutics targeting mast cell receptors and molecules involved in innate immunity are entering preclinical and clinical testing. Alongside, the portfolio of biologics is harboring the expansion of RNA therapeutics, which gained global recognition during the COVID‐19 pandemic due to RNA vaccines. Different types of RNA therapeutics, including those based on different non‐coding RNAs, are advancing to agency approval and market, thanks to improvements in molecule stability and delivery systems. In summary, the evidence presented in this position paper illustrates that precision medicine is becoming a goal shared between synthetic SMDs and biologics, both protein/antibody‐based and RNA therapeutics. We review the current state, unmet needs and opportunities within this evolving landscape, highlighting how small molecular species, both synthetic as SMDs and biologic in nature as RNA, can contribute to the precision medicine approach along with protein and antibody‐based biologics and cell therapies.

## Graphical abstract

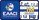

**Abbreviations**
A3A: adenosine A3
A3A‐R: adenosine A_3_ receptor
AC: adenylylcyclase
ACVR1: activin A receptor type 1
AD: atopic dermatitis
AE: adverse event
AHR: airway hyperresponsiveness
AhR: aryl hydrocarbon receptor
AK089514: annotated long non‐coding RNA
ALOX5: arachidonate 5‐lipoxygenase
ANRIL: antisense non‐coding RNA in the INK4 locus
ASO: antisense oligonucleotides
BTK: Bruton's tyrosine kinase
CASC7: cancer susceptibility candidate 7
CCR: chemokine receptor
CCR3: C‐C chemokine receptor 3
CFTR: cystic fibrosis transmembrane conductance regulator
cGAS: cyclic GMP–AMP synthase
CIndU: chronic inducible urticaria
circRNA: circular RNA
c‐kit: mast/stem cell growth factor receptor (SCFR), CD117
COPD: chronic obstructive pulmonary disease
COVID‐19: coronavirus disease 2019
CPK: creatine phosphokinase, also called creatine kinase, CK
CRNDE: colorectal neoplasia differentially expressed
CRTH2: chemoattractant receptor‐homologous molecule expressed on TH2 cells
CSU: chronic spontaneous urticaria
CXCR: C‐X‐C chemokine receptor
DYRK1A: dual‐specificity tyrosine‐(Y)‐phosphorylation regulated kinase 1A
ENST00000447867: Ensembl transcript ID for a specific lncRNA isoform
EoE: eosinophilic esophagitis
FDA: food and drug administration
FEV1: forced expiratory volume in 1 s
FLT3: fms‐like tyrosine kinase 3
GAS5: growth arrest specific 5
GATA3: GATA‐binding protein 3
GC: guanilylcyclase
GI: gastrointestinal
GR/NR3C1: glucocorticoid receptor/nuclear receptor subfamily 3, group c, member 1
HRH: histamine receptor H
ICAM‐1: intercellular adhesion molecule 1
IL: interleukin
IRAK: interleukin‐1 receptor‐associated kinase
JAK: Janus kinase
JPX: just proximal to XIST
KIT: KIT proto‐oncogene, receptor tyrosine kinase (CD117 or mast/stem cell growth factor receptor
LINC/PINT: Long intergenic non‐coding rna, p53 induced transcript
lncRNAs: long non‐coding RNAs
MALAT1: metastasis‐associated lung adenocarcinoma transcript 1
MAPK: mitogen‐activated protein kinase
MC: mast cell
MIR155HG: microRNA 155 host gene (also called BIC)
miRNA: micro RNA
MMP: matrix metalloproteinases
MRGPRX2: mas‐related G‐protein coupled receptor X2
mRNA: messenger RNA
NEAT1: nuclear paraspeckle assembly transcript 1
NF‐[x]B: nuclear factor kappa‐light‐chain‐enhancer of activated B cells
NLRP3: NOD‐, LRR‐ and pyrin domain‐containing protein 3
NO: nitric oxide
NR‐026690: transcript ID (RefSeq) for a lncRNA
PAH: pulmonary arterial hypertension
PDE: phosphodiesterases
PDGF: platelet‐derived growth factor
PDGFR: platelet‐derived growth factor receptor
PIK: PIM (provirus integration site for Moloney leukemia virus) kinase
PIK3CD/p110δ: phosphatidylinositol‐4,5‐bisphosphate 3‐kinase catalytic subunit delta
PsA: psoriatic arthritis
PSO: psoriasis
PTGDR2: prostaglandin D₂ receptor 2
PTTG3P: pituitary tumor‐transforming 3, pseudogene
PVT1: plasmacytoma variant translocation 1
RA: rheumatoid arthritis
RNA: ribonucleic acid
ROCK: Rho‐associated coiled‐coil containing protein kinase
S1P: sphingosine‐1‐phosphate
SGLT: sodium/glucose cotransporter
SGLT‐2i: sodium–glucose cotransporter 2 inhibitors
siRNA: small interfering RNA
SM: smooth muscle
SMDs: small molecule drugs
SNHG16: small nucleolar RNA host gene 16
SNHG5: small nucleolar RNA host gene 5
STAT: signal transducer and activator of transcription
STING: stimulator of interferon genes, also called TMEM173
SYK: spleen tyrosine kinase
T2D: type‐2 diabetes
TAZ: transcriptional co‐activator with PDZ‐binding motif
TBX: tromboxane
TBXA2R: thromboxane A_2_ receptor
TEC: tyrosine‐protein kinase Tec
THRIL: TNFα and HNRNPL related immunoregulatory lncRNA
TIMPS: tissue inhibitors of metalloproteinases
TLR: toll like receptor
TNF: tumor necrosis factor
TUG1: taurine upregulated gene 1
TYK 2: tyrosinkinase 2
UC: ulcerative colitis
URTI: upper respiratory tract infection
UTI: urinary tract infection
YAP: Yes‐associated protein

## 1 Introduction

Since the defining birth of aspirin following the synthesis of acetylsalicylic acid in 1897, the low molecular weight organic compounds described as small molecule drugs (SMDs) stand as the oldest pillar of pharmacological therapy [1]. For several decades, SMDs have been instrumental for treating chronic inflammatory diseases, which are diseases of high prevalence affecting large populations. In the last two decades, however, SMDs have been overshadowed by the development of protein‐based biologics and emerging cell‐ and CRISPR‐based therapies, which are powerful, targeted treatment options suited for a precision medicine approach [2, 3]. Yet, SMDs maintain their therapeutic relevance, representing roughly 90% of currently approved medicines and 40% of major pharmaceuticals' portfolios, the majority in oncology pipelines [4, 5].

In the first position paper issued in 2019, our Task Force reviewed SMD therapies in selected allergic diseases alongside biologics, highlighting their distinguishing characteristics [6]. Since then, several impactful changes occurred in the SMD field: some of the described classes have failed along the path to registration due to multiple reasons, including lack of clinical efficacy, unacceptable side effects, incorrect patient stratification, biomarker lacking sufficient specificity, and more [7, 8]. However, new achievements in this field were supported by a very significant leap in innovation, though less perceived than the rise of targeted therapies [9]. Advances in structural biology, generation of bioinformatics tools, widespread use of genetic sequencing and screenings to link genes with diseases, as well as the aid of machine learning and AI for SMD design, are among the critical factors behind such progress. Alongside, new paradigms in medicinal chemistry generated SMDs of novel design and mechanism of action [10, 11]. Moreover, innovative modes of drug delivery led to reassessment of SMDs excluded for systemic or off‐target effects or for bioavailability limitations [12, 13].

New insights on structural and mechanistic aspects of different classes of RNA, a molecule once considered undruggable for supposed lack of SMD binding sites, are supporting its therapeutic targeting or its use comparable to an SMD, with multiple approaches [14, 15]. Following mounting knowledge on the regulatory roles of noncoding RNA and mRNA biology in the last 50 years, culminating in the 2006 Nobel prize for the discovery of RNA interference, efforts on RNA‐based therapies expanded [16]. Early setbacks occurred when confronting multiple challenges, such as RNA degradation in plasma by nucleases, difficult cell penetration, and strong innate immune response. Chemical and sequence modification have currently improved the stability and decreased the immunogenicity of small RNA therapeutics; conjugation with ligands that increase cellular uptake and nanoparticle encapsulation improved their delivery. Although all these points need further improvement and surveillance for side effects [17, 18], the research effort and investment in oligonucleotide‐based therapies is now taking place again along with mRNA‐based technologies, with the Nobel Prize awarded to mRNA vaccines for COVID‐19 [19].

This position paper provides an update on the evolving landscape of synthetic SMD‐based therapies and presents preclinical evidence supporting the emerging use of several RNA species as a novel biological strategy for chronic allergic/inflammatory skin and lung diseases, with several affinities with SMDs.

New acquisitions in the current SMD‐based therapies and in the development of RNA‐based therapies will be described from three perspectives: first from the mechanistic view of targeted pathways, some of which are just emerging in preclinical settings; then, their clinical use with supporting evidence in chronic allergic/inflammatory skin and lung diseases; and lastly, their updated pharmacological features with indications and status of clinical investigations.

In conclusion, we will discuss the opportunities, limitations, and challenges that may support or hamper their therapeutic value along with, or in combination with, the protein and cell‐based targeted therapies.

## 2 Disease Mechanisms: Update on Established and Emerging SMD‐Targeted Pathways; Update on the RNA Species Working as SMDs and as Therapeutic Targets

### 2.1 JAK–STAT Pathways

**FIGURE 1:**
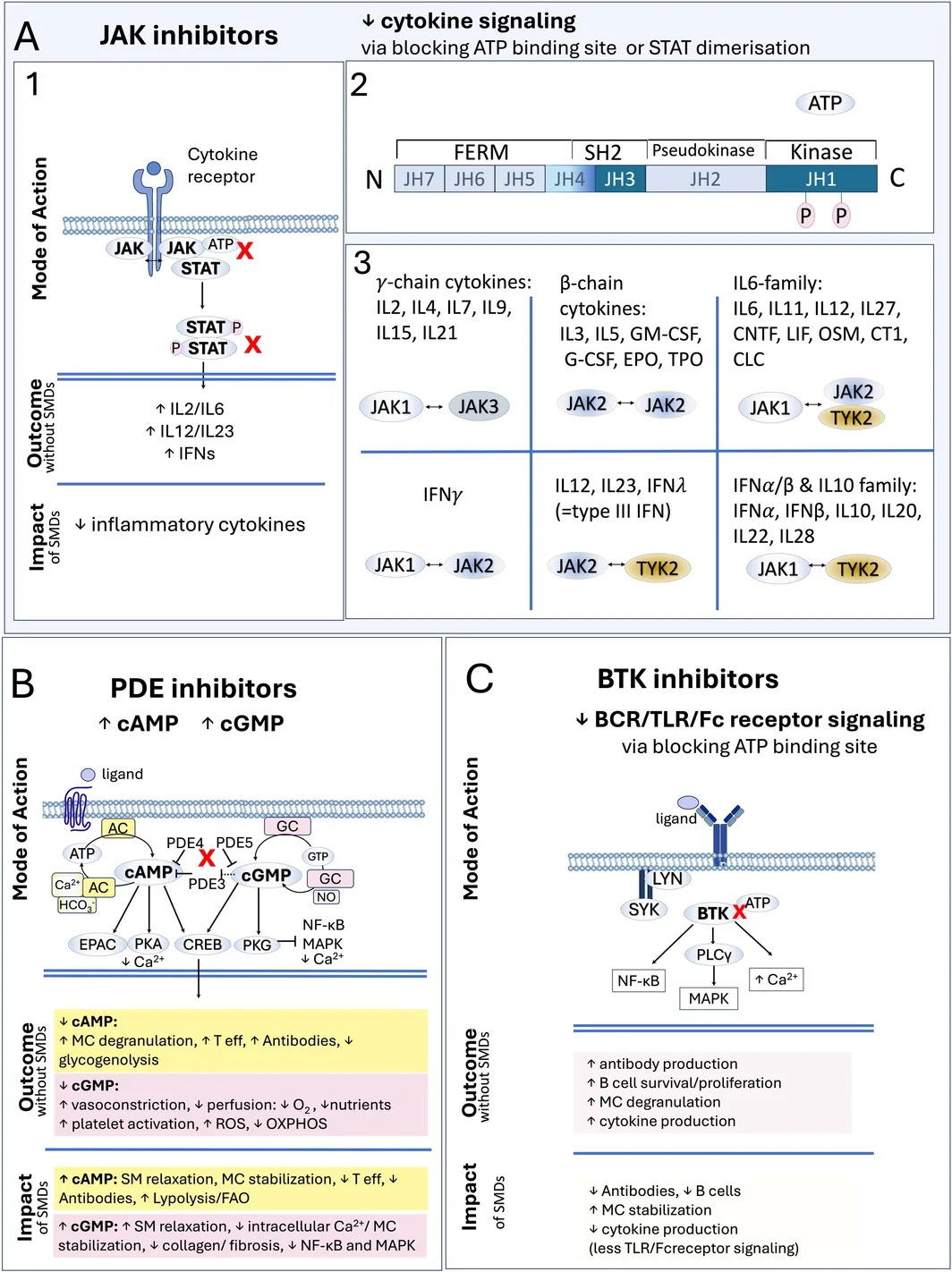
Mode of action of approved small molecular drugs. (A1) JAK inhibitors block the ATP‐binding site of Janus kinases (JAK1, JAK2, JAK3, and TYK2), preventing phosphorylation and dimerization of STATs, thereby halting cytokine‐driven gene transcription. (A2) Structural domains of JAK proteins (FERM, SH2, pseudokinase, kinase) and (A3) the cytokine receptor families/signaling pathways each JAK isoform is associated with. Approved JAK inhibitors—such as tofacitinib (JAK1/3), baricitinib (JAK1/2), upadacitinib (selective JAK1), and deucravacitinib (selective TYK2 via allosteric inhibition)—suppress immune cell activation and inflammation. (B) PDE inhibitors modulate intracellular signaling by preventing the breakdown of cyclic nucleotides, leading to cell type–specific physiological effects. PDE3 inhibitors primarily block cAMP hydrolysis (while being inhibited by cGMP), enhancing cardiac contractility and vasodilation; PDE4 inhibitors selectively increase cAMP in immune cells, reducing pro‐inflammatory cytokine production; PDE5 inhibitors prevent cGMP degradation in smooth muscle, promoting vasodilation and smooth muscle relaxation. (C) BTK inhibitors block the ATP‐binding site of Bruton's tyrosine kinase, disrupting B‐cell receptor and Fc receptor signaling. Approved agents inhibit B‐cell and myeloid cell activation, with covalent (e.g., ibrutinib, acalabrutinib) and non‐covalent (e.g., pirtobrutinib) binding modes.

Janus kinases (JAK) inhibitors disrupt inflammatory signaling preventing their phosphorylation, thereby blocking downstream STAT protein activation and signal transduction to the nucleus [20]. JAKs, nonreceptor tyrosine kinases (JAK1, JAK2, JAK3, tyrosine kinase 2 [TYK2]), mediate IFN and cytokine signaling (Figure 1A1). JAK1 [21] exhibits broad tissue expression, JAK2 [22] and TYK2 are enriched in monocytes while JAK3 [23] is primarily found in T cells within lymphoid tissues and mucosal linings, being crucial for T cell function and autoreactive T cell suppression [24]. Each JAK comprises seven homology domains (JHs) [25] (Figure 1A2), with JH1 possessing catalytic activity and an ATP‐binding site [26]. Notably, the FERM domain (JH5, 6, and part of JH4) plays a role in associating JAKs with cytokine receptors [27]. Inhibitors target the JH1 domain, blocking ATP binding and impacting the pseudokinase domain (JH2), thus hindering immune cell response to inflammatory stimuli and making them useful in immune‐mediated diseases (Figure 1A3).

Several generations of JAK inhibitors are established therapeutic tools in oncology and are registered for some inflammatory or auto‐immune diseases, including rheumatoid arthritis, psoriatic arthritis, juvenile idiopathic arthritis, ulcerative colitis [28, 29] and, as later discussed, currently under development for asthma. First‐generation molecules, targeting multiple JAKs (also defined as non‐selective inhibitors), exhibit broader side effects compared to second‐generation semi‐selective or selective inhibitors (so defined for blocking two or only one JAK pathway, respectively) (Table 1). Natural compounds, particularly polyphenols, inhibit JAK2 through complex mechanisms, offering anti‐inflammatory effects. For example, quercetin has been reported to directly bind JAK2 and inhibit JAK–STAT signaling [38]. JAK3 inhibitors, such as Z583 [68] are for autoimmune diseases and PF‐06651600 [69, 70] has demonstrated clinical efficacy in rheumatoid arthritis [71, 72, 73, 74] and is under investigation for alopecia areata and inflammatory bowel disease [72, 73, 74]. JAK inhibitor clinical efficacy is typically observed within 3–6 months of treatment, though some symptom improvement may occur within 1–2 weeks [75] as described for both topical ruxolitinib cream [76] and oral upadacitinib in atopic dermatitis [75, 76]. As side effects (Table 1), JAK inhibitors can induce anemia due to JAK2's role in erythropoietin signaling and may negatively impact thrombopoietin signaling and platelet homeostasis [77]. Lymphopenia and neutropenia are also potential side effects for JAK inhibitors. Because treatment increases the risk of herpes zoster reactivation, vaccination against herpes zoster is recommended prior to initiation (at least 4 weeks in advance in countries using a vaccine with live attenuated virus, an interval not necessary when using vaccines with inactivated virus); in patients without prior varicella exposure or vaccination, primary varicella immunization should be considered. Tofacitinib [78], a first‐generation JAK inhibitor, carries an increased risk of major cardiovascular events (heart attack, stroke, blood clots) and cancer, prompting FDA warnings for high‐risk individuals (persons over 65 of age, smokers, and people with a history of atherosclerotic and/or cardiovascular disease) [79]. JAK inhibitors have otherwise shown potential for hair regrowth, particularly with oral administration, possibly by suppressing inflammation and stimulating the anagen phase of the hair cycle [80]. They can also elevate serum lipid profiles, including LDL and HDL cholesterol, through mechanisms that are not fully understood [81]. Despite good tolerability, long‐term safety studies are recommended due to FDA concerns regarding systemic JAK inhibitors [47]. Systemic JAK inhibitor adverse events are dose‐dependent, with higher risks seen at increased doses (e.g., tofacitinib, upadacitinib, abrocitinib), supporting the use of the lowest effective dose to balance efficacy and safety [78, 82, 83].

**TABLE 1:** Current SMDs for skin and lung chronic allergic inflammatory diseases according to their targeted pathways, with main side effects (Paragraph 2).

SMD class and molecules	Primary targets	Side effects	Refs
**1. JAK inhibitors**			
*First generation JAK inhibitors*			
Tofacitinib (Xeljanz)	JAK1, JAK3	Infections (herpex zoster), anemia, neutropenia, lymphopenia, ↑ liver enzymes, ↑ LDL and HDL, nausea, diarrhea, headache, hypertension. Serious risks: upper respiratory tract infections (URTI), malignancies (lymphoma, lung), thrombosis, GI perforation, ↑ mortality CVD patients ≥ 50	[30, 31, 32, 33]
Baricitinib (Olumiant)	JAK1, JAK2	As above plus elevated creatinine in some patients	[30, 31, 32]
Ruxolitinib (Jakafi)	JAK1, JAK2	Anemia, thrombocytopenia, neutropenia, infections, metabolic: ↑ cholesterol, weight gain, headache, bruising. Serious risks: PML, infections, secondary malignancies (non‐melanoma skin cancer) withdrawal syndrome	[30, 31, 32, 34]
Oclatinib (Apoquel)	JAK1	Infections, GI: vomiting, diarrhea, hematologic: leukopenia, anemia, dermatologic: pyoderma, otitis externa	[30, 31, 32]
*Second generation JAK inhibitors*			
Filgotinib (Jyseleca)	JAK1	Headache, nausea, fatigue; ↑ lipids; infections (URTI, herpes zoster); ↓ sperm count	[30, 31, 32]
Upadacitinib (Rinvoq)	JAK1	URTI, herpes zoster, acne, nausea, cough; ↑ CPK, lipids; thrombosis, serious infections; ↑ cancer risk	[30, 31, 32]
Apeficitinib (ASP015K)	JAK3 > F JAK1/JAK2, TYK	Data limited, similar expected: cytopenia, ↑ CPK, infections, GI symptoms	[30, 31, 32]
Fedratinib (Inrebic)	JAK2	Wernicke's encephalopathy (boxed warning in patients with malnutrition or GI problems); nausea, vomiting, diarrhea, anemia, thrombocytopenia, ↑ liver enzymes	[30, 31, 32, 35]
Abrocitinib (Cibonqo)	JAK1 (JAK2)	Nausea, headache, acne; Herpes zoster; ↑ CPK, lipids, thrombocytopenia; rare: thromboembolism	[30, 31, 32]
Povorcitinib	JAK1	Cytopenia, ↑ CPK, nasopharyngitis, infections, acne, headache, nausea, ↑ risk for CVD evens and cancer	[30, 31, 32]
Momelotinib (Revolade)	JAK1, JAK2, ACVR1	Anemia, thrombocytopenia, peripheral neuropathy, diarrhea, fatigue, ↑ lipase/amylase (rare)	[30, 31, 32]
Decernotinib (Qinlock)	JAK3	Leukopenia, ↑ liver enzymes, lipids, infections.	[30, 31, 32]
Peficitinib	JAK3 > JAK1TYK2	Nasopharyngitis, herpes zoster, airway tract infections, cytopenia, ↑ liver enzymes, cholesterol	[30, 31, 32]
Ritlecitinib	JAK3 + TEC	Acne, headache, URTI, ↑ Liver enzymes	[30, 31, 32]
Lestaurtinib (CEP‐701)	FLT3, JAK2	Diarrhea, nausea, vomiting, cytopenia; discontinued poor tolerability	[30, 31, 32, 36]
*pan JAK inhibitors*			
Gusacitinib	JAK1, JAK2, JAK3, TYK2, SYK	↑ cholesterol, blood pressure, no serious AE reported	[30, 31, 32]
Delgocitinib	JAK1, JAK2, JAK3, TYK2	Nasopharyngitis, acne, headache, varicella eruption	[37]
*Natural compounds as JAK inhibitors*			
Tannins, e.g., Ellagitannins, proanthocyanidines, curcumin, resveratrol; polyphenols, e.g., epigallocatechin‐3‐gallate, catechin, fisetin, quercetin	JAK1, JAK2, STAT3 phosphorylation	Tannins, curcumin: GI irritation, hepatotoxicity (high intake); Resveratrol: GI symptoms, Polyphenols: generally safe; high dose: GI symptoms, ↑ liver enzymes	[38, 39, 40, 41, 42, 43, 44, 45, 46]
**2. Tyk2 inhibitors**			
Deucravacitinib	Allosteric Tyk 2	URI, herpes simplex, cold sores, mouth ulcers, acne, headache, hypertension, ↑ liver enzymes, ↑ CPK. Rare: malignancies, hypersensitv reactions	[30, 31, 32, 47]
Brepocitinib	Tyk2/JAK1	URTI, URI, GI symptoms	[30, 31, 32]
Ropsacitinib	Tyk2/JAK2	Headache, ↑ liver enzymes	[48]
QL‐1200186	Allosteric TYK2 inhibitor	Early‐stage development	[49]
*Natural Tyk2 inhibitors*			
Genistein (isoflavones in soy)	Tyk 2	GI symptoms, weak estrogenic activity; high dose: inhibits platelet activation	[50]
**3. Phosphodiesterase inhibitors**			
Ensifentrine	PDE3, PDE4	URTI, headache, cough, nasophyryngitis, diarrhea, dysgeusia	[51]
Roflumilast	PDE4	GI: nausea, diarrhea, abdominal pain, dyspepsia, weight loss. CNS: headache, insomnia, depression	[52]
Sildenafil	PDE5	Headache, flushing, hypotension, nasal congestion, rhinitis, dyspepsia, blurred vision (sildenafil), back pain (tadalafil), myalgia	[53, 54]
Tadalafil	PDE5		[54, 55]
Vardenafil	PDE5		[56]
Theophyllin	panPDE	GI: Nausea, vomiting, CNS: headache, insomnia, nervousness, cardio: tachycardia, arrhytmia, muscle: tremor, metabolic: hypokalemia, hyperglycemia (due to catecholamine release). Overdose: seizure, arrhytmia, intractable vomiting, rhabdomyolysis, metabolic acidosis	[57]
**4. BTK inhibitors**			
Ibrutinib	Irreversible, covalent	Atrial fibrillation, hypertension, cytopenia, GI symptoms, infections	[30, 31, 32, 58]
Fenebrutinib	Reversible, non‐covalent	Headache, URTI, GI upset, ↑ liver enzymes	[59]
Rilzabrutinib	Reversible, covalent	Diarrha, headache, ↑ liver enzymes	[60]
Remibrutinib	Irreversible	URTI, diarrhea, acne, fatigue	[61]
**5. MMP inhibitors**			
TIMPS	MMP10	Preclinical	[62]
MMP‐12 marmastat, prinomastat, rebimastat, batimastat	Broad‐spectrum inhibitors	Arthralgia, stiffness, GI: nausea, ↑ liver enzyme; preclinical	[63, 64]
Doxycycline hyclate		Mild GI symptoms, photosensitivity	[65]
MMP408	Selective MMP12	Preclinical	[66]
FP025	MMP12	Preclinical	[67]

Tyrosine kinase 2 (TYK2) is primarily expressed in monocytes and regulates cell growth, immunity, and cytokine/interferon signaling by activating STAT‐1, 3, 4, or 6 [84, 85, 86]. Upon ligand binding to heterodimeric receptors (TYK2‐associated chain and JAK1/2‐associated chain [87, 88]), TYK2 phosphorylates the receptor [89], creating STAT docking sites. STATs are then phosphorylated by TYK2 or JAK1/2, dimerize, translocate to the nucleus, and modulate gene expression [25]. Notably, TYK2 also negatively regulates STAT3 [90]. Compared to broad JAK inhibitors, TYK2 inhibitors offer a narrower spectrum of activity, particularly targeting IL‐12/23 driven diseases. Natural TYK2 inhibitors like genistein compete for the ATP binding site [91], similar to brepocitinib [92] and ropsacitinib [48]. Newer inhibitors, such as deucravacitinib, allosterically inhibit TYK2 by binding to the JH2 pseudokinase domain, preventing target protein phosphorylation.

### 2.2 Phosphodiesterases (PDEs)

PDEs comprise 11 superfamilies of enzymes (PDE1‐11) that regulate physiological processes via cyclic adenosine monophosphate (cAMP) and cyclic guanosine monophosphate (cGMP) [93]. Increased cAMP and cGMP levels lead to bronchodilation and anti‐inflammatory effects. PDEs (e.g., PDE3, PDE4, PDE5) [93] are implicated in chronic obstructive pulmonary disease (COPD), asthma and pulmonary arterial hypertension (PAH) and have been targeted therapeutically since the use of theophylline, followed by more selective inhibitors [94, 95, 96, 97] (Figure 1B, Table 1). In June 2024, the FDA approved ensifentrine (Ohtuvayre), a novel inhaled dual PDE3/PDE4 inhibitor, for COPD. Ensifentrine's approval was based on phase III studies (ENHANCE‐1 and ENHANCE‐2) [98, 99] involving moderate‐to‐severe COPD patients (FEV1 ~ 51%–52% predicted), with 69% of subjects being on LAMAs and 55% on combinations of LABAs/ICS, demonstrating significant improvements in FEV1 (90 mL increase), reduced symptoms and exacerbations, and enhanced quality of life compared to placebo. Ensifentrine's fast‐onset bronchodilation (PDE3) and inflammation suppression (PDE4) contributed to its efficacy, despite no anti‐inflammatory effect being observed in the approval studies. This approval was progressed due to the lack of side effects such as emesis and arrhythmias [94].

Other potential respiratory therapies include PDE4/7, PDE4/8, and PDE4/muscarinic antagonist/β2‐agonist combinations [97, 100].

### 2.3 Bruton Tyrosine Kinase (BTK)

BTK is highly expressed in myeloid and B cells, but not in T cells [101] and is essential for B cell development and signaling [102]. BTK possesses ATP, lipid‐, nucleotide‐ and zinc‐binding sites and is downstream of both B‐cell receptor and FcεRI signaling pathways [103] (Figure 1C). BTK mutations cause X‐linked agammaglobulinemia (XLA), a condition characterized by a severe deficiency of mature B lymphocytes and antibodies, leading to increased susceptibility to recurrent bacterial infections. BTK also activates myeloid innate immune cells via TLR signaling [102, 104], leading to NLRP3 inflammasome activity [105]. Ibrutinib, a first‐generation BTK inhibitor, suppressed NLRP3 activation [106] but was limited by significant toxicity caused by its covalent binding and off‐target activity against tyrosine‐protein kinase Tec (TEC), epidermal growth factor receptor (EGFR), interleukin‐2‐inducible T cell kinase (ITK) and other kinases, leading to severe adverse effects such as bleeding, cardiac arrhythmia, hypertension, infection, and skin toxicities [107] (Table 1). Second‐generation, highly selective BTK inhibitors (fenebrutinib, evobrutinib, tolebrutinib, remibrutinib, nibrutinib) are used in B cell malignancies [108] and are being investigated for multiple sclerosis. Importantly, these inhibitors block antibody synthesis and IgE‐mediated mast cell/basophil activation [50]. In addition, BTK signaling is functionally linked to integrin pathways, particularly VLA‐4 (α4β1 integrin [109]). Clinical trials on VLA‐4 antagonist in asthma were negative [110, 111, 112], but the pathway remains clinically relevant as the VLA‐4 inhibitor natalizumab is approved in multiple sclerosis [109, 110, 113].

### 2.4 Emerging Targetable Pathways

#### 2.4.1 Yes‐Associated Protein (YAP)–Transcriptional Coactivator With PDZ‐Binding Motif (TAZ) Pathway (YAP–TAZ)

**FIGURE 2:**
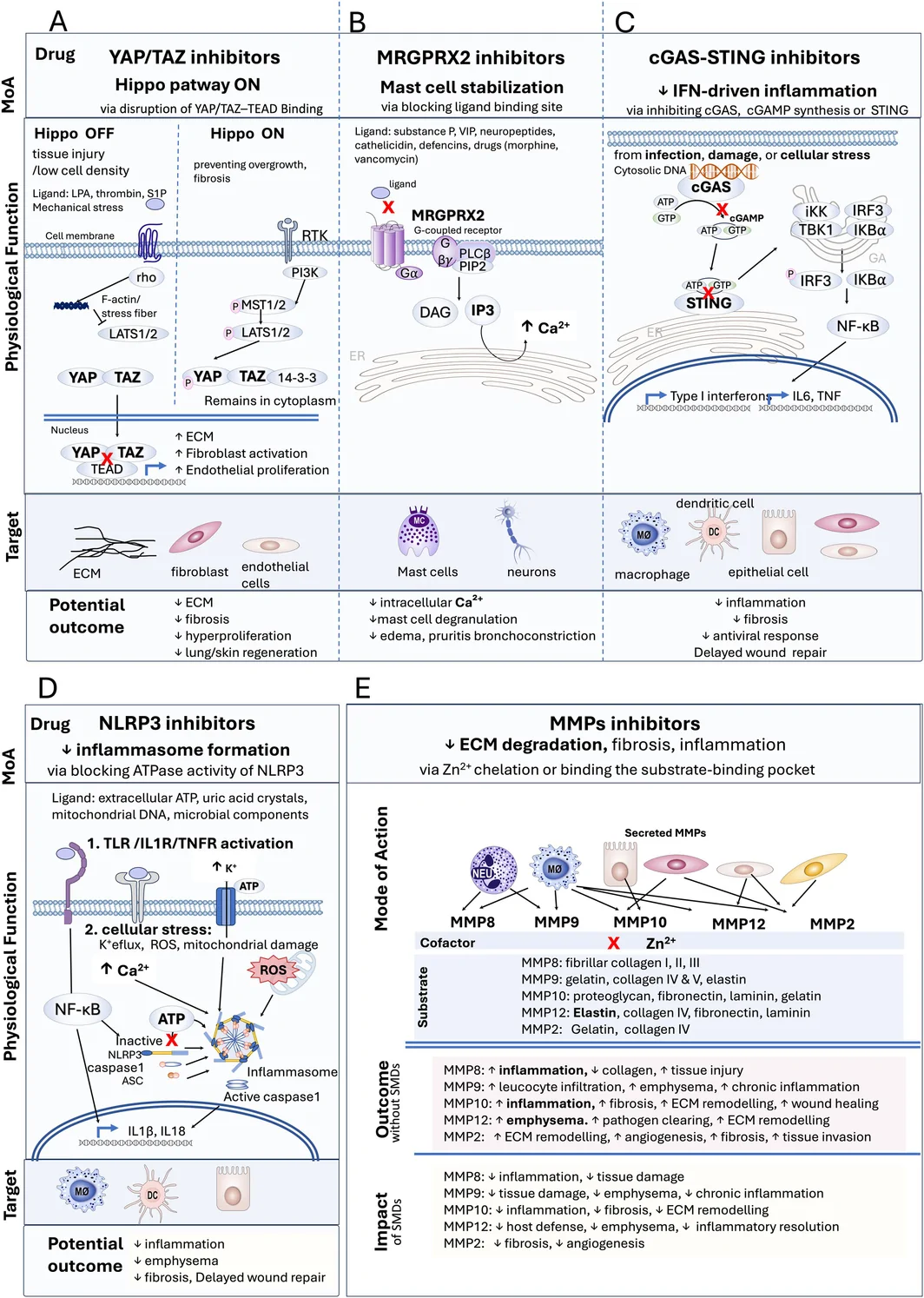
Mode of action of emerging therapeutical targets. (A) YAP/TAZ inhibitors primarily target fibroblast and endothelial cells. These inhibitors prevent YAP/TAZ from translocating into the nucleus, thereby preventing the expression of profibrotic genes. Additionally, endothelial cells are targeted, potentially leading to less vascular leakiness and preserving tissue function. (B) MRGPRX2 inhibitors antagonize the Mas‐related G protein‐coupled receptor X2 (MRGPRX2) on mast cells, blocking downstream signaling that normally increases intracellular calcium. This action effectively reduces mast cell degranulation and associated inflammatory responses. (C) cGAS‐STING inhibitors block the cGAS‐STING pathway, primarily by inhibiting the ability of cGAS to synthesize cGAMP or by directly inhibiting STING activation/oligomerization. This action prevents the downstream signaling cascade that normally activates transcription factors like IRF3 (leading to interferon production) and NF‐[x]B (leading to inflammatory cytokine production). They primarily target immune cells and infected cells, leading to attenuated inflammation but also a reduced antiviral response. (D) NLRP3 inhibitors suppress the NLRP3 inflammasome in myeloid cells by directly inhibiting NLRP3 protein activation, oligomerization, or its assembly into the inflammasome complex. This prevents the secretion of pro‐inflammatory cytokines such as IL‐1β and IL‐18, leading to reduced inflammation. (E) Secreted MMPs degrade extracellular matrix; their inhibition via zinc chelation limits inflammation, tissue damage, and fibrosis in chronic disease.

The expression of YAP or TAZ pathway is increased, beside in several cancers, also in many chronic inflammatory and fibrotic diseases including COPD and IPF [114] and in activated fibroblasts [115]. This pathway senses changes in matrix stiffness which is increased in many chronic inflammatory diseases (Figure 2A). More than 30 patents have been filed against this target, mostly related to oncology, and drugs such as IAG933 are now entering phase 1 clinical trials for advanced mesothelioma and other solid tumors [116, 117]. Considering the importance of matrix stiffness in many chronic allergic diseases, these drugs should be considered in these patients.

#### 2.4.2 Mas‐Related G‐Protein Coupled Receptor X2 (MRGPRX2)

The MRGPRX2 is a transmembrane protein which is predominantly expressed on mast cells (Figure 2B). MRGPRX2 can be activated by various cationic small compounds such as opioids, antibiotics, and neuropeptides and activation leads to mast cell degranulation mediating inflammatory and allergic responses. It is suggested that MRGPRX2‐mediated activation of mast cells may play a role in asthma exacerbations through mediators released by bronchial epithelial cells, eosinophils, and/or neutrophils [118].

#### 2.4.3 cGAS‐STING Pathway

The cyclic guanosine monophosphate‐adenosine monophosphate synthase (cGAS)‐stimulator of interferon genes (STING), cGAS‐STING pathway, discovered in 2013 [119], detects cytosolic DNA. cGAS binds DNA [120, 121, 122], producing cGAMP, which activates STING on the endoplasmatic reticulum (ER). Activated STING then translocates to the Golgi apparatus, where it activates TANK‐binding kinase (TBK)1, leading to IRF3 phosphorylation and dimerization [123], resulting in Type I and III IFN production [124, 125, 126]. TBK1 also activates NF‐[x]B, producing pro‐inflammatory cytokines (IL‐1β, IL‐6, and IL‐8) [125, 126] (Figure 2C). Normally, three‐prime repair exonuclease (TREX)1 prevents endogenous DNA activation [127]. However, damaged or foreign DNA [127], from sources like tumors, dead cells, mitochondria, bacteria ( *S. pneumoniae* , *M. tuberculosis* , and others), or viruses (Herpes simplex virus Type 1, Human adenovirus and others), can trigger this pathway [125]. Overactivation can lead to autoimmune disorders or cytokine storms [128, 129, 130], making cGAS‐STING a potential therapeutic target [131] for inflammatory diseases. In fact, in asthma mouse models [ovalbumin/house dust mite (HDM)], cytosolic double strand (ds)DNA activates cGAS, driving Type 2 allergic inflammation [132]; in asthmatics, serum mitochondrial (mt)DNA correlates with eosinophil counts. Moreover, STING agonists (diamidobenzimidazole/cGAMP) in HDM‐challenged mice show increased airway hyperresponsiveness, neutrophil recruitment, cell death, NET formation, dsDNA release, DNA sensor activation, and IFNγ/IL6/CXCL10 release [133], suggesting cGAS‐STING activation worsens asthma severity, particularly neutrophilic asthma. In COPD mouse models, cigarette smoke triggers release of dsDNA/mtDNA and other DAMPs from necrotic bronchial cells [134], inducing cGAS‐STING‐dependent lung inflammation, which is attenuated in cGAS/STING/IFNAR deficient mice [135]. Carbon black nanoparticles activate cGAS‐STING, driving IFN‐I‐dependent lung inflammation, also attenuated in cGAS/STING/IFNAR deficient mice [136]. cGAS/NF‐kB inhibitors (PF‐06928215/BAY 11‐7082) regulate PM2.5‐induced senescence via the cGAS/STING/NF‐kB pathway [137].

Inhibitors of cGAS‐STING [131] can be categorized by different mechanisms of action: (1) STING inhibitors (palmitoylation inhibitors [138, 139] cyclic dinucleotide pocket inhibitors [138], TBK‐1 inhibitors [140]); (2) cGAS inhibitors [141] (competitors for cGAS ATP or GTP binding sites; inhibitors of dsDNA binding to cGAS). Antimalarial drugs such as hydroxychloroquine, quinacrine, and suramin specifically bind to drug sites on the cGAS/dsDNA dimer to suppress cGAS activity [142].

#### 2.4.4 NLRP3 Inhibitors

The NLRP3 inflammasome, activated by pathogens and noxious stimuli, drives inflammation in chronic diseases via IL‐1β and IL‐18 release, leading to fibrosis [143, 144] (Figure 2D). The compound MCC950 (CRID3), a potent NLRP3 inhibitor (low nM efficacy), showed efficacy in animal models of allergic disease [145] but exhibited hepatic and renal toxicity [146]. Numerous NLRP3 inhibitors and a dual NLRP1/3 inhibitor are in clinical trials [147], while a dual NLRP1/3 inhibitor has shown efficacy in mice challenged with LPS, silica, and influenza A [148].

Targeting Toll‐like receptors (TLRs) involves receptor antagonism, signaling blockade, and protein degradation [149]. The effectiveness of hydroxychloroquine, an antimalarial drug targeting endosomal TLRs, in treating systemic lupus erythematosus (SLE) and rheumatoid arthritis (RA) suggests that suppressing TLR pathways may benefit other chronic inflammatory and allergic conditions. Targeting of specific TLRs and related pathways (e.g., with TLR7/8 inhibitors; IRAK4 kinase inhibitors and degraders [proteolysis‐targeting chimeras {PROTACs}]) is currently evaluated for AD and for RA, cutaneous LE (CLE), and SLE [150, 151, 152]. In a phase 2a study, CNTO3157, a TLR3 inhibitor, failed to protect asthma patients against respiratory events following an experimental rhinovirus challenge, while attenuated common cold symptoms in healthy subjects [153]. Overall, the complexity and redundancy of TLR pathways suggest that dual or multi‐targeted approaches will be more clinically successful.

#### 2.4.5 Matrix Metalloproteinases (MMPs)

MMPs is a family of at least 28 proteases involved in homeostatic tissue repair and immune modulation [154] (Figure 2E). Environmental triggers (e.g., pathogens, toxins, allergens) can promote lung tissue inflammation and remodeling through the imbalance between overexpressed MMPs and their endogenous inhibitors [155]. Several MMPs have been implicated in the pathophysiology of chronic lung inflammatory/fibrotic diseases and lung cancer [156, 157] and evaluated for therapeutic targeting [67, 158, 159]. The selective MMP‐12 inhibitors FP‐025 and MMP408 in experimental animals effectively blocked allergen‐induced airway responses and dampened the induced inflammatory and pro‐remodeling indices [67, 159]. In allergic asthma patients, multiple doses of oral FP‐025 effectively blocked the late‐phase sequelae following inhaled house dust mite challenge in subjects with mild eosinophilic asthma [67].

#### 2.4.6 Cell Senescence

Senotherapeutics act by inducing senescent cells' death (senolytic approach) or by blockage/modification of their metabolism and signaling governing the senescence‐associated secretory phenotype (SASP) (senostatic approach). We reviewed in our previous work senotherapeutic SMDs, their mechanisms and relative clinical trials, which currently are mainly evaluating safety and efficacy in single diseases [160, 161, 162].

#### 2.4.7 Glucagon‐Like Peptide‐1 Receptor (GLP1)

Glucagon‐like peptide‐1 receptor agonists (GLP1RAs) such as exenatide, liraglutide, lixisenatide, and semaglutide are extremely effective in treating metabolic syndrome and type 2 diabetes (T2D). As metabolic syndrome and T2D are often present in subjects with chronic allergic and inflammatory diseases, it has also been used in patients with psoriasis, asthma, and COPD [163]. Besides a reduction in metabolic disease/T2D, their clinical efficacy has also been associated with an improvement in immune and structural cell functions in the disease target organs attributed to weight loss [164]. However, there have been recent reports of adverse events such as skin thickness in patients with psoriasis and asthma, respectively [165, 166, 167]. Further studies need to be conducted, particularly in asthmatic patients who do not have metabolic syndrome as a comorbidity, for example, not just patients with obese asthma, to dissect whether these adverse events are drug‐ or disease sub‐phenotype related.

### 2.5 RNA as an SMD and as a Target: Non‐Coding RNA and RNA Modifications in Disease Pathophysiology

**FIGURE 3:**
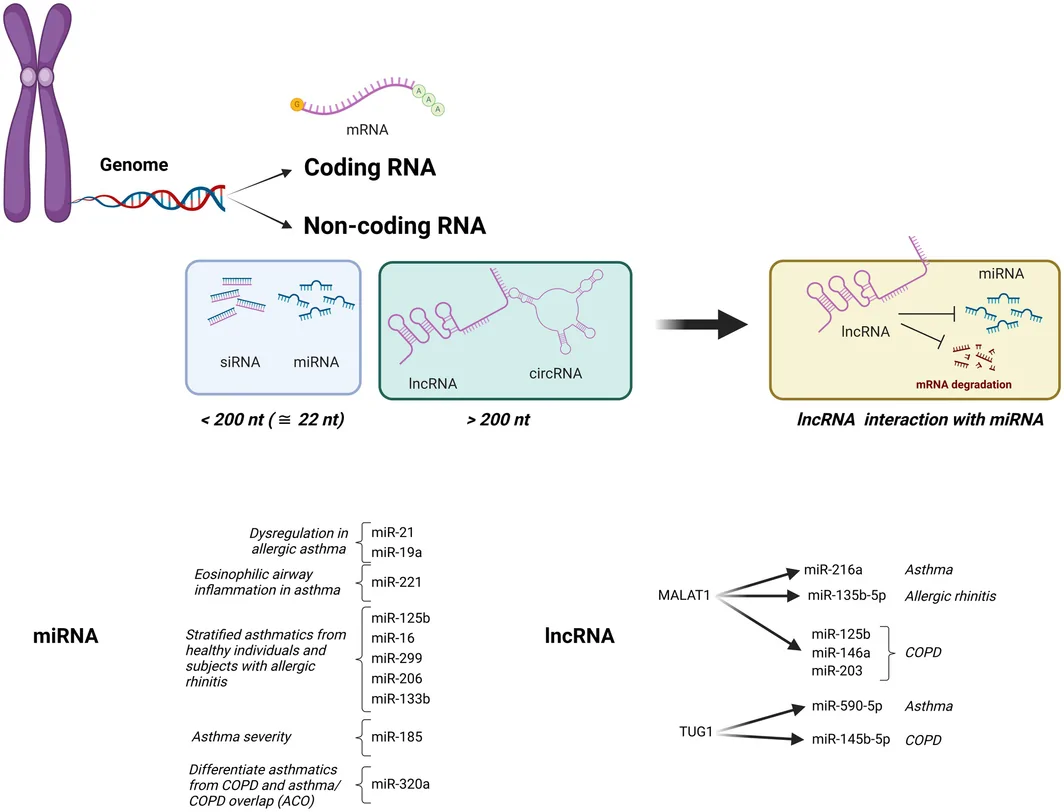
Types of regulatory lncRNA. As described by the ENCODE project, only 1%–2% of the human genome codes for proteins through messenger RNAs (mRNA); most transcripts constitute a heterogeneous pool of non‐coding RNA (ncRNA), critical in virtually all biological functions in homeostatic and disease processes. Non‐coding RNA control immune responses globally, by transcriptional and post‐transcriptional regulation. They comprise short and long ncRNA (below and above 200 nucleotides, respectively) and are generally divided into housekeeping ncRNA, involved in RNA biogenesis (not shown), and regulatory ncRNA; the latter include microRNA (miRNA), long non‐coding RNA (lnRNA), circular RNA (circRNA), and small interfering RNA (siRNA), with their characterization still growing in complexity.

Non‐coding RNA (ncRNA) control immune responses globally by transcriptional, post‐transcriptional and epigenetic regulation [168, 169] and their characterization is ever growing in complexity [170, 171, 172, 173, 174]. Regulatory ncRNAs include miRNA (average length of 22 nucleotides) and long non coding RNA (lncRNA) (average length above 200 nucleotides) (Figure 3). miRNAs and lncRNAs functions are connected, as lncRNA act as a sponge for miRNA by competitively binding to miRNAs to regulate the expression of target mRNAs, a process called competitive endogenous RNA (ceRNA) [175]. Both ncRNA species have gained recognition in chronic allergic and lung inflammatory diseases' mechanism and as potential biomarkers [176, 177]. Among miRNAs (Figure 3), the dysregulation of miR‐21 and miR‐19a has been reported in allergic asthma [178, 179, 180]. Epithelial and sputum miR‐221 was associated with eosinophilic airway inflammation in asthma [181], plasmatic miR‐125b, ‐16, ‐299, ‐206, and ‐133b stratified asthmatics from healthy individuals and subjects with allergic rhinitis [182], and miR‐185 was upregulated in eosinophils and sera from asthmatics compared to healthy controls, representing a promising predictor of asthma severity [183]. The expression of miR‐320a segregated asthmatic individuals from COPD and those with asthma/COPD overlap (ACO) [184].

As part of many preclinical data in asthma/COPD models, miR‐155 specifically inhibits IL‐13‐induced expression of eosinophilic chemokines CCL11 and CCL26 in bronchial epithelial cells [185] and miR‐487b can suppress the levels of mRNA and protein for IL‐33 during the differentiation of bone marrow‐derived macrophages, resulting in inhibition of IL‐33‐induced expression of antigen‐presenting and costimulatory molecules and proinflammatory mediators [186]. miR‐3202 reduced cigarette smoke‐induced MMP9, VEGFR, IFNγ, and TNF in a co‐culture of human bronchial epithelial cells and T cells [187].

Emerging data also identify lncRNAs in asthma mechanisms (Figure 3, Table 2). An exploration of lncRNA databases revealed 5 lncRNAs that could act as ceRNAs and may have an effect in asthma, including MALAT1, MIR17HG, CASC2, MAGI2‐AS3, and DAPK1‐IT1 [222]. Differential lncRNA expression was found in the peripheral blood of therapy‐resistant asthmatic children compared to controlled asthmatic children [223], as well as between asthma and control groups [224]. Importantly, different lncRNA/miRNA axes may play a role in allergic and lung inflammatory diseases. For instance, the lncRNA MALAT1 exhibited clinical implications in asthma (via miRNA‐216a) [190], allergic rhinitis (via miR‐135b‐5p) [206] and COPD (via miR‐125b, miR‐146a, and miR‐203) [216], while TUG1 contributes to asthma by promoting airway smooth muscle cell proliferation and migration (via miR‐590‐5p) [188] and airway remodeling in COPD (via miR‐145‐5p) [215].

**TABLE 2:** Main lncRNA and their miRNA targets implicated in allergic asthma, allergic rhinitis, and COPD pathophysiology.

lncRNA	Targeted miRNA	Refs
**1. Allergic asthma**		
TUG1	miR‐590‐5p	[188, 189]
MALAT1	miR‐216a	[190]
NEAT1	miR‐200a/b	[191, 192]
	miR124	
ANRIL	miR‐7‐5p	[193, 194, 195]
	miR‐98‐5p	
	miR‐125a	
THRIL	miR‐34a	[196]
	miR‐125b	
AK089514	miR‐125b‐5p	[197]
JPX		
PVT1	miR‐423‐5p	[198, 199]
	miR‐490‐5p	[200]
	miR‐149	
PTTG3P	miR‐192‐3p	[201]
GAS5	miR‐10a	[202]
SNHG16		
LINC/PINT	miR‐26a‐5p	[203]
CRNDE	miR‐33a	[204]
	miR‐181a	
	miR‐495	
CASC7	miR‐21	[205]
**2. Allergic rhinitis**		
MALAT1	miR‐135b‐5p	[206]
NEAT1	miR‐125a	[207]
	miR‐511	[208]
	miR‐21	[209]
	miR‐124	
	miR‐125a	
ANRIL	miR‐16‐5p	[210]
THRIL	miR‐125b	[211]
JPX	miR‐378 g	[212]
GAS5	miR‐21 and miR‐140	[213]
SNHG16	miR‐106b‐5p	[214]
**3. COPD**		
TUG1	miR‐145‐5p	[215]
MALAT1	miR‐125b	[216]
	miR‐146a	
	miR‐203	
PVT1	miR‐146a	[217, 218]
NR‐026690	—	[219]
ENST00000447867	—	[219]
MIR155HG	miR‐128‐5p	[220]
SNHG5	miR‐132	[221]

Chemical modifications of nucleosides through dedicated regulatory enzymes occur physiologically for all RNA species and form the epitranscriptome [225]. Epitranscriptomic changes respond to environmental cues; therefore, regulators' alteration or mutations change the decay or translation rates of hundreds of targeted RNAs, playing a pathogenic role identified in cancer and other diseases [226]. The methylation of adenosine (N6‐adenosine methylation, or m6A) is the most prevalent RNA modification and regulates the processing and fate of mRNAs [227]. Ongoing research is uncovering the immune epitranscriptome [228, 229] indicating m6A methylation as a regulator of T cell differentiation [230], of dendritic cells and NK cells activation [231, 232], and IgE‐mediated effector function in murine mast cells, including methylation‐dependent stabilization of IL‐13 mRNA [233]. Initial in silico epitranscriptomic analysis of U‐BIOPRED severe asthma PBMC GEO dataset identified two regulators (YTHDF3 and YTHDC1) associated with severe asthma and identified three different m6A RNA methylation patterns, based on differential expression of 27 m6A regulators; GO analysis indicated that these patterns could be related to the clinical phenotype of severe asthma [234]. Though still largely uncharacterized in lung inflammatory and allergic diseases, the epitranscriptome is increasingly explored as a disease biomarker source, with mounting preclinical evidence for its therapeutic targeting [235, 236, 237].

## 3 Clinical Update on SMDs and RNA‐Based Therapies in Skin and Lung Chronic Allergic and Inflammatory Diseases

**FIGURE 4:**
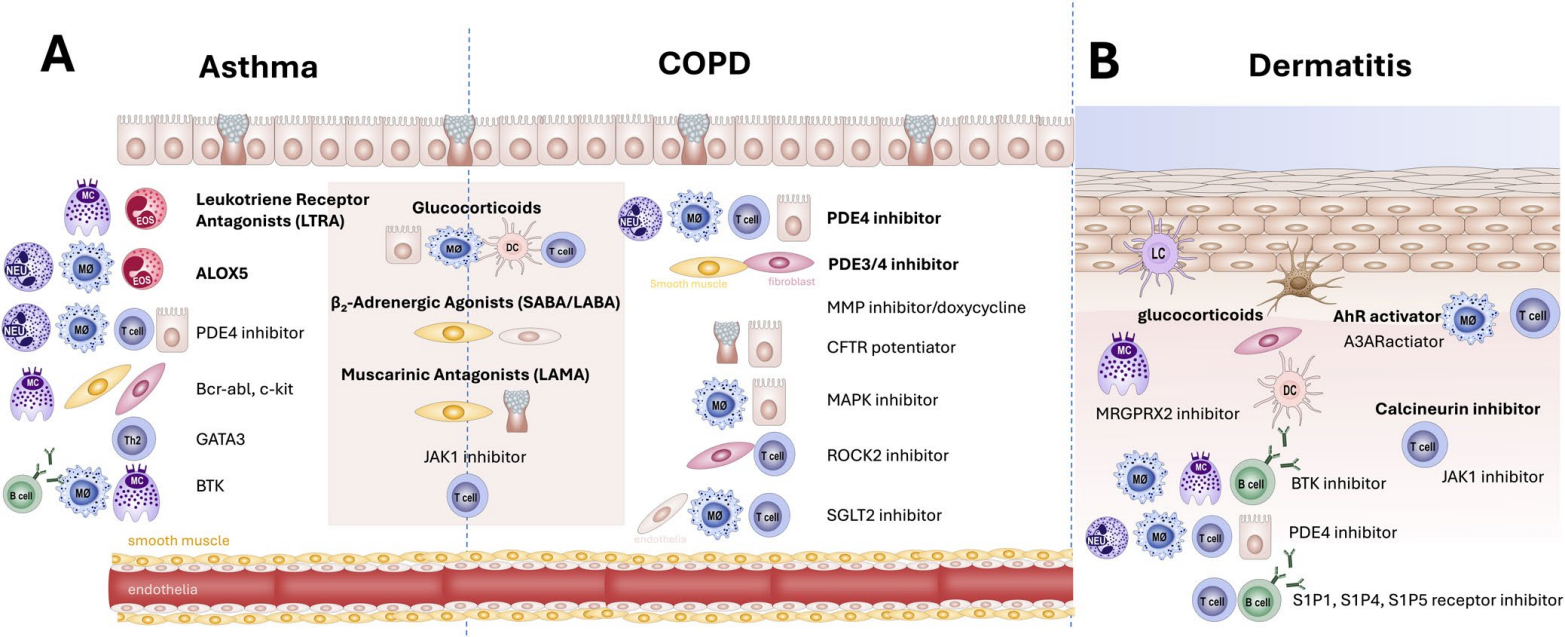
Current SMD strategies for asthma, COPD and skin inflammatory diseases. Approved drugs are shown in bold; investigational drugs in regular font. Glucocorticoids, SABA/LABA, and LAMAs are established treatments in both asthma and COPD, with PDE4 inhibitors primarily used in COPD, and LTRA and ALOX5 inhibitors in asthma. In contrast, current clinical trials target novel pathways: in asthma—BCR‐ABL/c‐KIT, GATA3, BTK, and PDE4; in COPD—MMP inhibitors, MAPK and ROCK2 inhibitors, SGLT2 inhibitors, and CFTR potentiators B. In atopic dermatitis, established therapies include topical glucocorticoids, calcineurin inhibitors, and the AhR activator. Emerging treatments under clinical investigation target BTK, PDE4, JAK1, MRGPRX2, S1P1/4/5 receptors, and A3A receptor pathways.

The SMDs targeting the pathways described in Par. 2 are part of current therapies or in clinical studies for main chronic skin and lung allergic/inflammatory diseases. To provide a more complete clinical update on SMDs in Table 3 we also included the pathways and relative SMDs described in our first position paper [6]. Figure 4 recapitulates the multiple cellular targets of all the SMDs listed in Table 3 according to major skin and lung diseases.

**TABLE 3:** Update on clinical studies for SMDs for skin and lung chronic allergic inflammatory diseases.

Target	Drug name	Indication	Route	Mechanism	Stage^a^
MMP9/12	AZD1236	COPD	Oral	Inhibitor	Discontinued
MMP12	FP‐025	Asthma, COPD	Oral	Inhibitor	Phase 1
IL‐2R	Rezperaldesleukin	AD	iv	rhIL‐2‐PEG conjugate	Phase 1b
		COPD		Antagonist	Discontinued
IL8R/CXCR2	Navarixin/MK‐7123	Asthma	Oral	Antagonist	Discontinued/terminated
	Danirixin	COPD	Oral	Antagonist	Discontinued/no efficacy
	AZD5069	Asthma	Oral	Antagonist	Phase 2 completed/pending
		COPD	Oral	Antagonist	Phase 2 completed/pending
TNF αR	Balinatunfib	PsO	Oral	Antagonist	Phase 2
CCR2b	AZD2423	COPD	Oral	Antagonist	Discontinued
CCR4	Zelnecirnon	AD	Oral	Antagonist	Discontinued
CRTH2/PTGDR2	Fevipiprant	Asthma	Oral	Antagonist	Phase 3 completed/no efficacy
		COPD	Oral		Terminated
	Timapiprant/OC459	Asthma	Oral	Antagonist	Phase 2 completed/pending
	AZD1981	Asthma	Oral	Antagonist	Phase 2 completed/pending
		COPD	Oral	Antagonist	Phase 2 completed/pending
	setipiprant	Asthma	Oral	Antagonist	Discontinued
	RG7185	Asthma	iv	Antagonist	Discontinued
TBXA2R	Seratrodast	Asthma	Oral	Antagonist	Approved (Japan, China, India)
Muscarinic receptors M1/2/3	Umeclidinium	Asthma	Inhal	Antagonist	Approved
	Glycopyrronium	Asthma	Inhal	Antagonist	Approved
	Oxitropium	Asthma	Inhal	Antagonist	Approved
		COPD	Inhal	Antagonist	Approved
Histamine receptors HRH 1/2/3/4	JNJ‐39758979	Asthma	Oral	Antagonist	Discontinued
	JNJ‐38518168—Toreforant	Asthma	Oral	Antagonist	Phase 2 No efficacy
PDE4	Roflumilast	COPD, AD, PsO	Oral	Inhibitor	Approved
	Cilomilast	Asthma/COPD	Oral	Inhibitor	Discontinued
	Aprelimast	PsA/PsO	Oral	Inhibitor	Approved
PDE3/PDE4	Ensifentrine (Ohtuvayre)	COPD	Inhal	Inhibitor	Approved
5‐lipoxygenase/ALOX5	Zileuton	COPD	Oral	Inhibitor	Discontinued
		Asthma	Oral	Inhibitor	Approved
	MK‐0633/Setileuton	Asthma	Oral	Inhibitor	Discontinued
		COPD	Oral	Inhibitor	Discontinued
	PF‐04191834	Asthma	Oral	Inhibitor	Discontinued
Glucocorticoid receptor GR/NR3C1	Triamcinolone acetonide	Asthma	Inhal	Agonist	Approved
		COPD		Agonist	Approved
	Mometasone furoate	Asthma	Inhal	Agonist	Approved
		COPD	Inhal	Agonist	Approved
		AD	Topical	Agonist	Approved
PIK3CD/p110d	Nemiralisib/GSK2269557	Asthma	Inhal	Inhibitor	Discontinued
		COPD	Inhal	Inhibitor	Discontinued
S1P1, S1P4, S1P5 receptor	Etrasimod	UC	Oral	Inhibitor	Approved
		EoE		Inhibitor	Phase 2
		AD		Inhibitor	Phase 2/3
	Ponesimod	PsO	Oral	Inhibitor	Phase 2
CFTR	Icenticaftor—QBW251	COPD	Oral	Stimulator	Phase 2
A3A‐R	Piclidenoson/CF101	PsO	Oral	Agonist	Phase 3 (not started yet)
Aryl hydrocarbon receptor (AhR)	Tapinarof	PsO	Topical	Agonist	Approved (topical)
KIT, PDGFRA and PDGFRB	Imatinib	Asthma	Oral	Inhibitor	Phase 2
JAK1	Abrocitinib (Cibinqo)	AD	Oral		Approved
	Upadacitinib	AD/PsA	Oral	Inhibitor	Approved
	Filgotinib	RA (no asthma, COPD, AD)	Oral	Inhibitor	Approved
	Apeficitinib	RA (no asthma, COPD, AD)	Oral	Inhibitor	Approved
	Povocitinib	Asthma	Oral	Inhibitor	Phase 2
		CSU		Inhibitor	Phase 2
	AZD4604	Asthma	Inhal	Inhibitor	Phase 1/2
	GDC‐0214	Asthma	Inhal	Inhibitor	Phase 1 completed mild asthma
	GDC‐4379	Asthma	Inhal	Inhibitor	Phase 1 completed mild asthma
	AZD0449	Asthma	Inhal	Inhibitor	Phase 1
JAK1/2	VR588	Asthma	Inhal	Inhibitor	Early Phase 1
	Baricitinib	Alopecia Areata, AD, RA	Oral	Inhibitor	Approved
	Ruxolitinib	AD	Oral	Inhibitor	Approved
JAK3	PF‐06651600	CsU	Oral	Inhibitor	Phase 2
	Decernotinib/VX‐509	RA	Oral	Inhibitor	Discontinued
JAK1/2/3	Tofacitinib	PsA	Oral	Inhibitor	Approved
TYK‐2	Deucravacitinib (Sotyktu)	Plaque psoriasis	Oral	Inhibitor	Approved
		PsA	Oral	Inhibitor	Phase 3
	Zasocitinib	PsO/PsA	Oral	Inhibitor	Phase 3
	Ropsacitinib	PsO		Inhibitor	Phase 2
TYK2‐JAK1	Brepocitinib	PsA	Oral	Inhibitor	Phase 2
	TTL‐018	RA	Oral	Inhibitor	Phase 2
TYK2‐JAK1/2/3	Gusacitinib	AD	Oral	Inhibitor	Phase 2
	Delgocitinib	AD	Topical	Inhibitor	Approved
	Peficitinib	PsO/RA		Inhibitor	Approved
	Frevecitinib (KN‐002)	Asthma	Inhal	Inhibitor	Phase 2b initiated
	TD‐8236	Asthma	Inhal	Inhibitor	Phase 1 completed
JAK‐SYK	Gusacitinib	AD	Oral	Inhibitor	Phase 2 terminated
MAPK	A2D7624	Asthma	Oral	Inhibitor	Phase 2
	PF‐03715455	Asthma	Oral	Inhibitor	Discontinued
	CMF6297	COPD	Oral	Inhibitor	Phase 1/2 suspended
	PF03715455	COPD	Oral	Inhibitor	Phase 2
	Losmapimod	COPD	Oral	Inhibitor	Phase 2
	Dilmapimod (SB681323)	COPD	Oral	Inhibitor	Phase 2, no efficacy
	Losmapimod	COPD	Oral	Inhibitor	Phase 2, no efficacy
GATA‐3	SB010	Asthma	Inhal	DNAzyme	Phase 2a
	SB011	AD	Topical	DNAzyme	Phase 2a
	SB012	UC	Oral	DNAzyme	Phase 2a
BTK	Remibrutinib	Asthma	Oral	Inhibitor	Phase 2 terminated
		CSU		Inhibitor	Phase 3
		Food allergy		Inhibitor	Phase 2
	Rilzabrutinib	Asthma	Oral	Inhibitor	Phase 2
		CSU	Oral	Inhibitor	Phase 2
		AD	Oral	Inhibitor	Phase 2
	Fenebrutinib	CSU	Oral	Inhibitor	Phase 2
	Ibrutinib	Food allergy	Oral	Inhibitor	Phase 2
	Acalabrutinib	Food allergy	Oral	Inhibitor	Phase 2
Rho‐kinase	Belumosudil	PsO	Oral	Inhibitor	Approved only for cGVHD
IRAK	SAR444656	AD	Oral	Degrader	Phase 2
MRGPRX2	EVO756	CIndU, CSU, AD	Oral	Inhibitor	Phase 2
	INCB000262	CSU	Oral	Inhibitor	Phase 2 paused
SGLT‐2i	Dapaglifozin	T2D	Oral	Inhibitor	Anti‐inflammatory effects observed; improvement in asthma
	Empaglifozin	T2D	Oral	Inhibitor	

^a^ Stage may be different according to countries where the studies have been initiated.

### 3.1 SMDs in Chronic Urticaria (CU) and Atopic Dermatitis (AD)

Chronic urticaria is characterized by the onset of pruritic hives with/without angioedema for ≥ 6 weeks [238]. It can be triggered by physical stimuli (chronic inducible urticaria, CIndU) but mostly occurs without any recognizable cause (chronic spontaneous urticaria, CSU) [239]. Along with guideline therapy and monoclonal antibodies in clinical studies [240], therapies with several oral SMDs are under investigation in subsets of CU (Table 3). In particular, JAK inhibitors approved for autoimmune arthritis (e.g., tofacitinib) have also been administered successfully to CSU patients [241, 242] and other molecules (e.g., porvocitinib) are currently under investigation in CSU.

A single PDE4 inhibitor is currently approved for dermatitis: crisaborole, a topical agent indicated for mild‐to‐moderate atopic dermatitis [243]. The BTK inhibitors fenebrutinib [244] and rilzabrutinib [245] are being studied for chronic skin diseases. A phase II clinical trial showed fenebrutinib as effective in decreasing CSU activity in antihistamine (AH)‐resistant patients, although moderate abnormalities in liver parameters were also observed. Remibrutinib showed significant clinical improvements and a good safety profile in subjects with AH‐resistant CSU [246] and in phase 3 trials for CSU, showing improved symptom control [50]. Mas‐related G protein‐coupled receptor X2 (MRGPRX2) drives IgE‐independent activation of dermal MC in subjects with CSU and other dermatosis [247]. Currently, clinical trials with small molecule receptor antagonists EVO756 (Evommune) and EP262 (Incyte) in CSU/CIndU/AD are underway (ClinicalTrials.gov); no clinical studies in asthma/COPD patients are documented yet.

For AD, several SMDs have been recently added to therapeutics or are in clinical studies. Currently, three oral JAK inhibitors are available for AD [248]. Ruxolitinib is a topical JAK1 and JAK2 inhibitor approved by the FDA for adolescents and adults with mild‐to‐moderate AD [249]. Despite its topical administration, ruxolitinib was able to reduce T2 chemokines in sera from AD patients. Upadacitinib and abrocitinib are indicated in adolescents and adults with moderate‐to‐severe AD, whereas baricitinib is approved in adults with the same indication [248]. Although the initial studies showcased reduced chemokines and cytokines production, recent studies found these changes to be more subtle. Treatment for 48 weeks with upadacitinib demonstrated increased serum IgE and TARC/CCL17 levels; the latter increased also in an observational, investigator‐driven analysis of the three JAK inhibitors in a 52‐week treatment, along with increased PARC/CCL18, CTACK/CCL27 and IL‐18 levels in the skin [250, 251]. Gusacitinib (ASN002), an oral JAK/Syk inhibitor, demonstrated rapid efficacy and improved epithelial barrier function in phase 2 studies for moderate‐to‐severe atopic dermatitis, with significant anti‐inflammatory effects and improved patient quality of life [252, 253]. JAKi inhibitors trigger a more rapid improvement of eczema than monoclonal antibodies (within days vs. within weeks), but their effect also disappears shortly after discontinuation [252]. JAKi can induce dose‐dependent and rapidly reversible lymphopenia, neutropenia, anemia and dyslipemia, and increase the risk of HZV infections [252] (Table 1).

Other SMD being developed for AD include Apremilast, an oral PD4 inhibitor which induced a clinical benefit of eczema, a peripheral reduction of Th22 and Th17 markers, and an improvement of epidermal hyperplasia [254]; Zelnecirnon, an oral selective antagonist of CCR4, a chemokine receptor expressed on Th2 cells binding TARC/CCL17 [255]. In a phase 1b trial, this drug showed clinical benefits of eczema, while decreasing inflammatory mediators related to Th2, Th1, Th17 and Th22 cells in sera from AD patients [255].

Recently, a novel approach has been tested in the treatment of AD. Rezperaldesleukin, a PEG conjugation of rhIL‐2, increased regulatory T cells (Tregs). In a phase 1b trial in AD, an 83% improvement in EASI was obtained, and after treatment discontinuation the clinical effects are maintained for 36 weeks [256].

### 3.2 SMDs in Chronic Inflammatory Pulmonary Diseases

#### 3.2.1 JAK Inhibitors

New oral JAK‐inhibitors are in early development for asthma and COPD (Table 3). Inhaled formulations, including selective compounds GDC‐4379 and GDC‐0214, aim at reducing systemic effects and are currently in development for asthma. Inhaled formulations in phase 1 or 2 studies have so far been well tolerated; they have shown improvements in clinical parameters along with dose‐dependent reductions in FeNO and sputum eosinophils in asthmatic patients [257, 258, 259, 260, 261] but further and larger studies are needed to establish long‐term safety and efficacy [257, 258, 259, 260].

#### 3.2.2 BTK Inhibitors

Emerging agents in the treatment of chronic lung diseases are BTK inhibitors such as ibrutinib, which reduce pathogenic immune activation. In the lung, BTK signaling contributes to macrophage activation, mast cell degranulation and B‐cell driven inflammation. Inhibitors optimized to limit off‐target effects, such as evobrutinib, have shown to suppress B cell activation and inflammation in preclinical models [262, 263], with ibrutinib shown to reduce mast‐cell mediated itch and inflammation [263]. In a phase 2 study, asthma patients experienced fewer asthma control loss events and symptom improvements with both high and low doses of rilzabrutinib [245].

#### 3.2.3 MMP‐Inhibitors

Increased MMP activity (resulting from increased MMP/TIMP ratio) has been detected within the airways of patients with asthma and COPD following exacerbations and in more severe disease. Given their pro‐inflammatory and pro‐fibrogenic properties, several MMPs (e.g., MMP‐9 and MMP‐12) have been proposed as potential targets in these respiratory conditions [154]. However, given the involvement of MMPs in several physiological processes, unsurprisingly non‐selective MMP‐inhibition has been associated with serious adverse events [154]. Within the respiratory field, a few studies with targeted MMP‐inhibitors have been conducted in humans. Other studies in COPD patients and asymptomatic smokers show potentially different roles of MMP‐12 (from detrimental to protective) across study populations [264, 265], possibly caused by the interplay between MMP‐polymorphisms and environmental factors [266, 267, 268]. Similarly, MMP‐12 was associated with several key characteristics of asthma including type 2 inflammation and airway hyperresponsiveness (AHR) [269] as well as higher levels of MMP‐12 corresponded with more severe disease [159, 269]. In subjects with asthma, increased levels of MMP‐9 associated with the magnitude of allergen‐induced late asthmatic response (LAR) [270, 271], and selective MMP‐12 inhibition (with oral FP‐025) attenuated the allergen‐induced sequelae of the LAR (large and small airways physiology, allergen‐induced airway AHR and FeNO levels) [67, 272]. The dualistic properties of MMPs, possibly related to genetic polymorphisms, warrant further research into their complex mechanistic pathways and interactions as well as the potential role of selective MMP‐inhibitors in the treatment of pulmonary diseases.

### 3.3 ncRNA Targeting in Chronic Lung and Allergic Diseases: Preclinical Evidence

#### 3.3.1 miRNA Mimics and Antagomirs

Both mimics of miRNA or synthetic antagonists to miRNA, called antagomirs (Table 4), have been studied as miRNA‐modulating agents in cellular or mouse models of asthma and COPD [273]. Antagomirs of miR‐9 restored steroid sensitivity to airway hyperresponsiveness (AHR) in a steroid‐resistant mouse model exposed to IFNγ and LPS by increasing protein phosphatase 2A and glucocorticoid receptor nuclear translocation [274], while antagomir of miR‐106a suppressed airway inflammation, goblet cell hyperplasia, subepithelial fibrosis, and AHR in an allergic murine model by increasing IL‐10 expression [275, 276].

**TABLE 4:** Terminated clinical trials for selected SMDs (January 2015 – August 2025).

Disease and SMD	Drug targets	ClinicalTrials.gov ID
**1. Asthma**		
MK‐1029	CRTH2 inhibitor	NCT01656395
Remibrutinib	BTK inhibitor	NCT03944707
PF‐03715455	p38 MAPK inhibitor	NCT02219048
Fiboflapon	FLAP inhibitor	NCT00850642
Fevipiprant	CRTH2 antagonist	NCT03052517
RPT193	CCR4 antagonist	NCT05935332
CHF 6532	PGD2 antagonist	NCT04049175
Urticaria		
THB001	Wild type KIT inhibitor	NCT05510843
**2. COPD**		
PF‐03715455	p38 MAPK inhibitor	NCT02366637
**3. Atopic dermatitis**		
RPT193	Anti CCR4	NCT05399368
Gusacitinib	SYK/JAK inhibitor	NCT03654755
Ovatodiolide	Increased ROS formation	NCT04220411
FRTX‐02	DYRK1A inhibitor	NCT05382819

In asthma models, transfection of either miR‐146a or miR‐146b mimics in airway smooth muscle cells reduced cyclooxygenase 2 and IL‐1β expression induced by pro‐inflammatory cytokines, suggesting potential usefulness of these miR mimics in asthma [277]. miR‐126, miR‐145, and miR‐21 antagomirs [17, 18, 278] significantly reduced airway hyperresponsiveness, eosinophil recruitment, mucus hypersecretion and Th2 cell activation in a house dust mouse model. Of relevance to this effect, miR‐145 was shown to be involved in the maintenance of the Th1/TH2 balance in asthma by interfering with Runt‐related transcription factor 3 (RUNX3) through studying the effect of a miR‐145 mimic on asthmatic CD4+ T‐cells [279]. miR‐19a mimic enhanced cell proliferation of bronchial epithelial cells from severe asthma through targeting TGF‐β receptor 2 mRNA; conversely, repressed expression of miR‐19a by an antagomir increased SMAD3 phosphorylation through TGF‐β receptor 2 signaling and abrogated bronchial epithelial cell proliferation [280].

In COPD models, antagomir for miRNA‐570 in COPD small airway epithelial cells reduced abnormality associated with cellular senescence such as a reduction in the SASP phenotype and restoration of sirtuin levels [281]. miR125a/b antagomirs increased mitochondrial antiviral signaling, IFNβ and IFNλ protein and reduced viral replication in COPD primary bronchial epithelial cells from healthy and COPD subjects; on the other hand, their mimics reduced the induction of antiviral cytokines and increased virus titres and therefore appear of relevance in enhanced antiviral immunity to influenza virus in COPD [282]. In cigarette smoke extract‐exposed human bronchial epithelial cells, miR‐145‐5p mimics reduced the expression of Kruppel‐like 5 factor (KLF5), p53, cleaved caspases 3/9, the nuclear translocation of NF[x]B p65 subunit and the secretion of pro‐inflammatory cytokines [283]. Through the reduction in activation of PPARγ activation and sensitizing toll receptor‐like signals, miR‐27‐3p antagomir reversed pro‐inflammatory pathways and reduced CS‐induced neutrophilia and macrophage lung infiltration and BAL levels of IL1β, TNFα, and IFNγ [284].

#### 3.3.2 lncRNA Modulation

The airway smooth muscle is not only contractile but can also contribute to the inflammatory process in asthma and COPD [285, 286]. There was increased expression of LINC00882, LINC00883, and PVT1 in primary airway smooth muscle cells treated with dexamethasone [287]. Using siRNA against PVT1, the importance of this lncRNA in controlling both proliferation and IL‐6 release of airway smooth muscle cells from patients with severe asthma was shown [288]. Knock‐down of GAS5 using an siGAS5 reduced PDGF‐BB induced BDNF expression and ASMC proliferation and decreased airway hyperresponsiveness in asthmatic rats, which was reversed by miR‐10a inhibitor [202]. Thus, in asthmatic rats GAS5 regulated the airway smooth muscle proliferation via miR‐10a/BDNF [202]. Another lnc, taurine‐upregulated gene 1 (TUG1) promoted the proliferation and migration of airway smooth muscle cells through sponging miR‐590‐5p/FGF‐1 [188]. It was also proposed that four upregulated lncRNAs (RP11‐46A10.4, LINC00883, BCYRN1, and LINC00882) acted as miRNA “sponges” for 4 miRNAs (miR‐150, ‐371‐5p, ‐940, ‐1207‐5p). The lncRNA BCYRN1 promoted the proliferation and migration of rat airway smooth muscle cells from an ovalbumin‐asthma model via upregulating the expression of canonical or classic transient receptor potential (TRPC1) [289]. The TUG1 lncRNA can act through sponging several different miRNAs such as miR‐145‐5p, which silences the protease DUSP6. shRNA that targets TUG1 inhibited airway inflammation and remodeling in a cigarette smoke‐induced COPD model [215]. In macrophages, TUG1 regulates Th2 cell differentiation via miR29c/B7‐H3 axis [290]. Furthermore, lncMEG3 can act as a ceRNA to regulate the expression of RORɣ through miRNA‐17, affecting the balance of Treg/Th17 in asthma [291].

## 4 Pharmacology: Status on Past, Present, and Upcoming SMDs and RNA Therapies

### 4.1 JAK Inhibitors

Along with successful applications in cancer and chronic inflammatory diseases such as IBD and AR, the current use of JAK inhibitors in skin inflammatory diseases, including AD, represents a critical step towards SMD‐based targeted therapies for other chronic allergic and inflammatory disorders (Table 3). Still, kinome‐targeted SMD development is hampered by multiple isoforms/substrates, context‐dependent signaling, and pathway redundancy/compensation, often leading to limited efficacy or clinically relevant adverse effects [270]. For JAKs, focusing on specific family members within defined cytokine‐receptor subsets improves target selectivity, a virtue once chiefly associated with mAbs. In respiratory disease, inhaled JAK‐inhibitor formulations (e.g., GDC‐0214, GDC‐4379, and the pan‐JAK frevecitinib) are being tested in asthma/COPD, but human data remain limited to Phase 1c/2 studies with no Phase 3 trials ongoing and not approved [292]. Accordingly, long‐term safety—especially in older adults—must be established before clinical adoption.

### 4.2 Discontinued SMDs

Discontinued drugs in asthma, COPD, urticaria, and atopic dermatitis (Table 4) largely targeted type‐2 inflammatory pathways—such as CRTH2/PGD2, CCR4, BTK, SYK/JAK, p38 MAPK, FLAP, and DYRK1A—but despite biomarker effects, most failed due to limited clinical efficacy or safety concerns while RNA‐based approaches are mostly in preclinical stages or entered phase 1/1c [293].

### 4.3 RNA‐Based Therapies

Only a small fraction of approved biologics are RNA therapeutics, most of which target rare diseases such as Duchenne muscular dystrophy, spinal muscular atrophy, and cytomegalovirus retinitis [17], but based on their approval there is very significant growth in research and industry investment for this technology [294, 295]. In the case of chronic treatments, specific attention will be needed to oversee potential long‐term side effects.

#### 4.3.1 Oligonucleotide‐Based Therapies: Working as SMDs but Targeting RNA

The mechanisms of RNA interference by ncRNA species are different and can be exploited as a therapeutic approach (Table 5), by interfering with pathologic gene profiles caused by ncRNA‐driven alteration of the targeted transcripts [296].

**TABLE 5:** Selected RNA‐based therapeutic strategies.

Targeting	Therapeutic RNA species	Outcomes
**1. Complementary RNA**		
siRNA	Synthetic, double‐strand RNA molecule, length 19–21 nt	Antagonizes mRNA function: targets a sequence of *specific* mRNA for its degradation through RNA interference
miRNA	Endogenous single‐stranded short RNA molecules	Conveys miRNA function: targets *multiple* mRNA targets for degradation
miRNA mimic	Synthetic RNA duplexes with one strand duplicating an endogenous miRNA	Mimics the endogenous functions of the miRNA of interest
Antisense oligonucleotide (ASOs)	Synthetic noncoding single stranded RNA with full complementary to coding sequence of targeted RNA or miRNA (antimiRs, antagomirs)	Antagonizes *specific* mRNA/miRNA function
**2. mRNA**	protein‐coding RNA molecule	Different effector functions according to expressed protein

##### 4.3.1.1 Small Interfering RNA (siRNA)

Targeting miRNA and lncRNA forms the basis of oligonucleotide‐based therapies, using modified or unmodified nucleotides. In addition to miRNA and lncRNA, siRNA are ncRNAs specifically acting only on one or a group of mRNA targets, causing gene silencing at the post‐transcriptional level. In particular, siRNA induces gene silencing by sequence‐specific cleavage of mRNA, resulting in the inhibition of protein synthesis [189]. The only silencing RNA that has reached the clinical trial stage for respiratory disease targets SYK kinase, which is linked to the release of pro‐inflammatory mediators, with gene silencing of SYK kinase by siRNA in bronchial epithelial cells decreasing inflammatory mediators IL‐6, ICAM‐1 and NO [297, 298]. An inhaled siRNA, EXcellair, had a Phase 2 trial in asthma but this was discontinued [299].

In the murine model, effective delivery of siRNA to activated cells has been achieved [300] and on this result, applications of targeted siRNA delivery to macrophages, T cells and dendritic cells for asthma have been proposed [301].

##### 4.3.1.2 Antisense Oligonucleotides (ASO)

Several ASO's for asthma and COPD have entered clinical trials but none have yet passed beyond Phase 2 trials. ASO targeting Adenosine A1 receptor [302], combined bc and CCR3 [303, 304, 305], IL‐4/IL13 receptor α chain [306], for asthma and combined PDE4B/4D/7A [307] for COPD have been studied.

##### 4.3.1.3 Delivery of Oligonucleotide‐Based Therapies

The fact that oligonucleotides can be delivered directly to the airways and lungs by inhalation provides some advantages in terms of the therapeutic response to the use of interference RNA in respiratory diseases: apart from delivering directly to the site of disease ensuring therapeutic target engagement, delivery by inhalation reduces the potential systemic side‐effects, particularly in the treatment of asthma. Enhancing the stability of RNA so that there is cell membrane penetration and endosomal escape remains a challenge [308]. The commonly used delivery systems for aerosol delivery include polymer‐based, lipid‐based or peptide‐based, with lipid‐based systems commonly used, including liposomes and nanoparticles. Exosomes, which are extracellular vesicles containing genetic material including mRNA and miRNA, proteins, and lipids, are also being explored as a delivery system [309, 310].

#### 4.3.2 RNA as SMD: From Vaccines to Allergy Immunotherapy (IT)

The COVID‐19 pandemic led to the accelerated approval of mRNA vaccines for widespread use, even though the first human trials investigating RNA vaccines for an infectious disease (rabies) began in 2013 [311]. The design of RNA vaccines requires that the genome of the infectious agent has been sequenced so that pathogen antigen‐specific encoding mRNA can be transferred into cells, where the RNA is translated by the cell into antigenic proteins. This should then induce an antigen‐specific adaptive immune response, which generates the memory lymphocytes that protect against infection by the pathogen in the future. Typically, the RNA is encapsulated in lipid nanoparticles (LNPs) that protect the RNA strands and help their absorption into the cells [312]. All the LNPs approved to date for clinical use consist of four lipids: an ionizable cationic lipid, cholesterol, a phospholipid (helper lipid), and a polyethylene glycol (PEG)‐conjugated lipid (PEGylated lipid) [313]. While the classic LNPs composition allows for RNA absorption into most organs and tissues, albeit with predominant localization to the liver, significant research efforts are examining how modifications of lipid composition can help to selectively target LNPs to other organs such as the lungs and spleen [314, 315]. In addition to lipid composition modifications, incorporation of protein macromolecules and antibodies into LNPs may help cellular targeting [316]. For example, adding a mannose moiety supports active transport of the LNPs into dendritic cells and improves intradermal delivery [317, 318]. Targeting the central nervous system has been shown using a neurotransmitter conjugated with an ionizable lipid [319], while conjugation of functionalized PEGylated lipid with antibodies such as anti‐CD4 allows for T cell targeting [320]. In addition to infectious diseases, RNA vaccines are being extensively examined for their potential as immunotherapeutic approaches for treating tumors [321]. Rather than using pathogen genes, tumor RNA vaccines use genes encoding for tumor‐specific antigens. RNA based immunotherapy offers numerous advantages including allowing antigen‐presenting cells to present multiple epitopes thereby stimulating a broader range of T‐cell responses and there is no risk of integration into the host genome [322]. An interesting finding with RNA vaccines is that high levels of IgG4 are sometimes induced, perhaps due to the high antigen concentration and repeated vaccination [323], which might have a role in preventing immune over‐activation, similar to successful allergen‐specific immunotherapy as IgE‐induced effects are inhibited by IgG4. Another interesting allergen immunotherapy approach being evaluated is the use of virus like particles that incorporate prokaryote RNA and peanut allergens. Importantly, immunogenicity and vaccine effectiveness were influenced by RNA dependent activation of toll‐like receptors, especially TLR‐7 [324].

## 5 Conclusions

Collectively, the evidence shows that a targeted therapeutic approach can increasingly be achieved by multiple strategies, including the new synthetic SMDs born from evolving concepts and innovative technologies in medicinal chemistry [7]. The FDA approval of kinase inhibitors in chronic allergic conditions of the skin and initiation of clinical trials in asthma and COPD, where none is approved yet, underscores how a deeper knowledge of human kinome provides critical understanding of its role and position within disease subtypes, ultimately providing a first SMD option for specific patient populations. As previously noted, however, the long‐term safety profile is yet to be fully established for toxicity (including drug–drug interactions), effects of immunosuppression and cancer surveillance, especially in the elderly.

As part of the heterogeneous toolbox of biologics, a growing body of noncoding RNA medical products are positioned to become another therapeutic pillar [18] in the foreseeable future. In allergic diseases, RNA‐based drugs have largely yet to advance beyond early‐stage clinical trials, but this is likely a future path to new treatments. Several antisense‐ and RNAi‐based RNA therapeutics have recently reached regulatory approval and market, after facing a “valley of death” following the initial development in early 2000 [325, 326]. Implementation for several rare diseases is now established [17] and clinical studies are now approaching major diseases, such as cancer [327], metabolic diseases [328], cardiovascular diseases [329] and in preclinical studies, autoimmune diseases [330, 331]. This marks a pivotal step forward for this approach, where advances in technology, growing financial investment, and an expanding set of targetable diseases could converge to deliver breakthrough therapies—similar to the trajectory of monoclonal antibodies, which required more than two decades to achieve approval and widespread clinical use.

As other biologics, though, RNA therapies currently remain costly. This represents a major distinctive factor from synthetic SMDs and contributes to a significant gap in patient access to therapies [4, 332]. Nevertheless, increasing manufacturing scale, platform standardization, and advances in delivery and dose optimization may reduce costs over time. In parallel, the intrinsic flexibility and target specificity of RNA‐based approaches may make them particularly well suited for precision and personalized applications—such as inherited immunodeficiencies or immunodysregulated pathways—potentially more so than synthetic small molecules, despite their higher upfront costs. Compared to all types of biologics, SMDs' relatively higher side effects rate hampers in part the advantage of lower cost, while biologics with their targeted mechanism are less prone to systemic side effects, but carry a much higher cost for patients.

Given the unprecedented diversity of molecules and approaches developing for both biologics and synthetic drugs we describe in this and previous position papers [6, 160, 333], one unmet need that should be prioritized is the identification and validation of flexible treatment strategies for precision medicine that would be both effective and affordable. Besides facilitating patients' access to biologics promoting alternatives such as biosimilars, preclinical and clinical research should focus on evaluating more closely how combination therapies interact and how to exploit this potential: for example, the use of SMDs at lower doses, curbing side effects while adding treatment efficacy or allowing a longer spacing for biological dosing. Multiple preclinical studies with inhibitors of different kinase classes (such as JAK, MAPK) show a decrease in steroid resistance in PBMC and airway epithelial cells from severe asthma and COPD patients [270]. Accordingly, clinical trials should also focus on assessing combination therapies on disease control or remission, evaluated in specific patient subpopulations as part of precision medicine, and including cost containment as one of primary outcomes.

### 5.1 Methodological Approach

Our methodological approach combined structured expert workstreams with a coordinated consensus process. On initial consensus on the topic gathered at the TF business meeting (virtual, 18.07.2023), an initial excel spreadsheet with main sections and subsections was circulated. Each member was assigned a defined thematic area and independently conducted a targeted review of the relevant evidence. These contributions were then integrated into a unified draft, which all members jointly evaluated for accuracy, coherence, and completeness. Through iterative group discussions and a final collective revision, the panel reached consensus on the final content, ensuring that the position paper reflects a comprehensive, balanced, and rigorously synthesized assessment of current knowledge.

## Author Contributions

The topic for this fourth TIPCO paper was chosen unanimously; specific parts were drafted by authors' subgroups: abstract, introduction, conclusion: C.S.; basic pathophysiology: F.R.‐W., I.M.A., G.C., M.J., Z.D., L.B.; RNA: O.P., C.B.‐V., R.B., L.O., M.J., G.N., L.C.; clinical update: K.F.C., G.C., I.E.‐G.; E.K., Z.D., M.J.; pharmacology: I.M.A., F.L.‐S., M.Z., F.A.R., B.V.E., I.E.‐G.; glossary, Table 1, Figures 1 and 2: F.R.‐W.; Table 2, Figure 3: C.B.‐V., O.P.; Tables 3 and 4: B.V.E., F.A.R.; Table 5, C.S. The first draft was assembled during an ad‐hoc hybrid meeting (Vietri sul Mare [SA], Italy, November 2024), edited by F.R.‐W. and C.S. and submitted by F.R.‐W. after final read and approval by authors. C.S. coordinated and structured the manuscript and assembled the final draft. Manuscript editing/formatting for publishing by F.R.‐W. All authors contributed to writing and critically revised the manuscript for the intellectual content. All authors approved the final version of the manuscript.

## Funding

This Position Paper was supported by the European Academy of Allergy and Clinical Immunology (EAACI) under the EAACI Taskforce Immunopharmacology (TIPCO) and Immunology Section, 40314, 2023‐2025.

## Conflicts of Interest

F.R.‐W. has no conflicts of interest regarding the subject of this manuscript. F.R.‐W. is lead inventor of EP2894478, has served as an investigator, speaker, and/or advisor for Biomedical Int R&D, Allergy Therapeutics, and Bencard, and is founder of ViaLym. I.M.A. reports speaker or consultant honoraria and/or served on advisory boards at AstraZeneca, GSK, Kineset, and Sanofi. He has received investigator‐led funding for studies from GSK, Sanofi, and Sysmex that are outside of the submitted work. I.M.A. is currently Head of the Asthma Section in EAACI. L.B. reports during the last 3 years speaker or consultant honoraria and/or served on advisory boards at AstraZeneca, Chiesi, Sanofi, and Phargentis. M.J. reports during the last 3 years speaker or consultant honoraria and/or served on Advisory boards at Takeda, Pfizer, AstraZeneca, BerlinChemie Menarini, Chiesi, Novartis, Viatris, Sanofi, CSL Behring, and Swixx Pharma. G.C. reports unrestricted educational and/or research grants and/or lecture fees and/or grants for travel and accommodation to participate in scientific meetings from: Astra Zeneca, Alfasigma (Biofutura merged in it), Boehringer‐Ingelheim, Chiesi Farmaceutici, Enfarma, GSK, Menarini Group, NeoPharmed‐Gentili, Piam‐Bruschettini, Vivisol. K.F.C. has received honoraria for participating in Advisory Board meetings of GSK, Astra Zeneca, Novartis, Roche, Sanofi, Merck, Trevi, Nocion, Shionogi, and Rickett‐Beckinson and has been renumerated for speaking engagements for GSK, Sanofi, Novartis, and AZ. He holds research grants from GSK and Merck, paid to his institution. He is on the Scientific Advisory Board of The Clean Breathing Institute, supported by Haleon. Z.D. received speaker or consultant honoraria and/or served on advisory boards at: Aclaris Therapeutics (previously Biosion), Arrowheadpharma, Cascador Health (previously Galenushealth), Curovir, Foresee Pharmaceuticals, GlaxoSmithKline, Hippo‐Dx, Pleuran SRO, QPS‐Netherlands, Sanofi‐Genzyme‐Regeneron, TPG. Travel grant received from Airsonett AB and ALK. During her assignment as guest professor, institutional grants from Sanofi were received by KUL, Leuven, Belgium. She serves/served as scientific editor to MedNet (2016–2024), associate editor for Respiratory Medicine (2004–ongoing) and Allergy (2018–2024), and as Chair of the Asthma Expert Panel at EUFOREA (2020–2024). I.E.‐G. has received honoraria for lectures and advisory activities from HAL Allergy, Allergy Therapeutics, Inmunotek, LetiPharma, Diater, ALK, Allergopharma, Chiesi, Gebro, GSK, AstraZeneca, Sanofi, Novartis, Abbvie, Celltrion, and Viatrix. L.O. reports research grants from Chiesi, Reckitt, and Fonterra, and participation in speaker bureau for Nestle, Yakult, Reckitt, and Abbott. O.P. received research grants from MINECO, Ministerio de Ciencia e Innovación, Inmunotek S.L., Novartis, and AstraZeneca and fees for giving scientific lectures or participation in Advisory Boards from: AstraZeneca, Pfizer, GlaxoSmithKline, Inmunotek S.L.L, Novartis, SanofiGenzyme and Regeneron. The other authors declare no conflicts of interest.

## Data Availability

The authors have nothing to report.
